# Prognostic value of CD146 in solid tumor: A Systematic Review and Meta-analysis

**DOI:** 10.1038/s41598-017-01061-3

**Published:** 2017-06-26

**Authors:** Ping Zeng, Hai Li, Pei-Hua Lu, Li-Na Zhou, Min Tang, Chao-Ying Liu, Min-Bin Chen

**Affiliations:** 1 Department of Radiotherapy and Oncology, Kunshan First People’s Hospital Affiliated to Jiangsu University, 91 Qianjin Road, Kunshan, 215300 Jiangsu Province China; 2 Department of Medical Oncology, Wuxi People’s Hospital of Nanjing Medical University, No. 299, Qingyang Road, Wuxi, 214023 Jiangsu Province China

## Abstract

CD146, also known as melanoma cell adhesion molecule, was initially identified as a marker of melanoma progression and metastasis. Recently many clinical studies investigated overexpression of CD146 predict poor prognosis of solid tumor, however, the results was inconclusive, partly due to small numbers of patients included. This present meta-analysis was therefore performed utilizing the results of all clinical studies concerned to determine the prognostic value of CD146 expression in solid tumors. Relevant articles were identified through searching the PubMed, Web of Science and Embase database. In this meta-analysis, 12 studies involving 2,694 participants were included, and we drew the conclusion that strong significant associations between CD146 expression and all endpoints: overall survival (OS) [hazard ratio (HR) = 2.496, 95% confidence interval (95% CI) 2.115–2.946], time to progression (TTP) (HR = 2.445, 95% CI 1.975–3.027). Furthermore, the subgroup analysis revealed that the associations between CD146 overexpression and the outcome endpoints (OS or TTP) were significant in Mongoloid patients and Caucasian patients, as well in patients with lung cancer and digestive system cancer. In conclusion, these results showed that high CD146 was associated with poor survival in human solid tumors. CD146 may be a valuable prognosis predictive biomarker; nevertheless, whether CD146 could be a potential therapeutic target in human solid tumors needs to be further studied.

## Introduction

CD146, also referred to as MUC18, MCAM, Mel-CAM, S-Endo-1 and P1H12 antigen^1^, a transmembrane glycoprotein belonging to the immunoglobulin superfamily that functions as a Ca^2+^ independent adhesion molecule^2^, was first identified as a melanoma-specific cell adhesion molecule. CD146 was a 113-kD membrane glycoprotein that contains five immunoglobulin-like domains, a transmembrane region, and a short cytoplasmic tail^3^. Early research indicated that CD146 is expressed on vascular endothelium, smooth muscle and other cells in normal tissue, and mediates cation-independent adhesion through interactions with an unidentified ligand on the surface of various cells^4^. Subsequent research manifested CD146 was a multifunctional molecule that participates in several physiological and pathological processes involving in development, immunity, and angiogenesis^5^. CD146 regulated development of the nervous system, kidney, and retina^6–8^. Knockdown of CD146 protein expression impeded vascular development whereas overexpression of CD146 in zebrafish induces sprouting angiogenesis^9^. In recent years, cumulated evidence indicated that CD146 overexpression significantly correlates with the progression, angiogenesis, metastasis of some malignant tumors which was observed in esophageal cancer, melanoma, gallbladder adenocarcinoma, ovarian carcinoma, prostate cancer^10–16^. However, in oral mucoepidermoid carcinoma, CD146 expression was greater in intermediate/high grade tumors, weaker in patients with local recurrence, regional and distant metastasis^17^. CD146 was found downregulate in breast cancer and pancreatic cancer progression^18, 19^. Recently, An increasing number of studies suggested that CD146 is highly expressed in solid tumors, including hepatocellular carcinoma^20^, leiomyosarcoma^21^, esophageal squamous cell carcinoma^22^, lung cancer^23–25^, colorectal cancer^26^, clear cell renal cell carcinoma^27^, gastric cancer^28^, gallbladder adenocarcinoma^13^, breast cancer^29^, epithelial ovarian cancer^30^. These conspicuous indications on the role of CD146 in cancer indicated that the transmembrane glycoprotein would be further considered as a potential marker for outcome of cancer patients.

Plenty studies showed that increased CD146 expression in tumor tissues was associated with poor survival of patients with various cancer types. However, the results of those individual studies were not comprehensive. Therefore, we performed this comprehensive meta-analysis aiming to clarify the prognostic value of CD146 in solid tumors and to support that the protein may be a potential therapeutic oncotarget.

## Materials and Methods

### Publication search

According to the Preferred Reporting Items for Systematics Reviews and Meta-Analyses guidelines^31^, this present meta-analysis was performed. A comprehensive literature search was implemented by using the electronic databases PubMed, Embase, and Web of Science databases (up to July 20, 2016) with the search terms: ‘CD146’, ‘MCAM’ and “cancer”/“tumor”/“neoplasm”/“carcinoma” and the following limits: Human, article in English. Wholly potentially eligible studies were retrieved and their bibliographies were carefully scanned to identify other eligible studies and extra studies were identified by a hand search of the references cited in the original studies. When multiple studies of the same patient population were identified, we incorporated the published report into the largest sample size.

### Inclusion criteria

To be qualified for presence in this meta-analysis and data extraction, studies had to: (a) assess CD146 expression in predicting prognosis in cancer, (b) offer hazard ratios (HRs) with 95% confidence intervals (CIs) or enable calculation of these statistics from the data presented, (c) sort CD146 expression into “high” and “low” or “positive” and “negative”.

### Exclusion criteria

Exclusion criteria were: (a) literatures published as letters, editorials, abstracts, reviews, case reports and expert opinions; (b) experiments performed *in vitro* or *in vivo*, but not based on patients; (c) articles without the HRs and 95% CI, or not dealing with overall survival, disease-free survival, or the K-M survival curves; (d) studies in which the follow-up duration was shorter than 3 years.

### Data extraction

All data included in the present meta-analysis were extracted independently and carefully by two reviewers using a standardized form. Disagreement was resolved through independently extracting data from the original article by the third author, and consensus was reached by discussions. The meta-analysis of CD146 expression was based on two outcome endpoints: OS (overall survival), TTP (time to progression). DFS (disease-free survival) and TTR (time to recurrence) that similar in meaning were combined to use a unified prognostic parameter: time to tumor progression (TTP). Several different parameters, if reported, were extracted from each paper, including the first author’s surname, publication year, country of origin, number of patients analyzed, types of measurement, and score for CD146 assessment, cut-off values to determine CD146 overexpression, OS, TTP. The main features of these eligible studies were summarized in Table 1. The multivariate HR was extracted to assess prognostic value of CD146 expression. For the articles in which prognosis was plotted only with the Kaplan-Meier curves, the Engauge Digitizer V4.1 was then used to extract survival data, and the estimation of the HRs and 95% CIs were calculated with Tierney’s method^32^. All studies were assessed via Newcastle-Ottawa Scale (NOS). The quality scores ranged from 6 to 9, suggesting that the methodological quality was high.

**Table 1:** Characteristics of studies included in the meta-analysis.

Author	Year	Country	Case	Disease	Method	Cut off value	Endpoints	N OS
Jiang G^20^	2016	China	120	hepatocellular carcinoma	qRT-PCR	averaged 2^−ΔCT^ value	OS, TTP	8
Zhou Y^21^	2015	China	36	leiomyosarcoma	IHC	scores of (++) or (+++)	OS, TTP	8
Li Y^22^	2014	China	63	esophageal squamous cell carcinoma	IHC	positive cells ≥25% and/or scores ≥2	OS	6
Zhang X^23^	2013	China	118	non-small cell lung cancer	IHC	score of 3–6 (range of 0–6)	OS	8
Tian B^26^	2013	China	1080	colorectal cancer	IHC	scored as 1+ or 2+	OS	8
Oka S^24^	2012	Japan	183	lung adenocarcinoma	IHC	cellular membrane staining ≥10%	OS	9
Feng G^27^	2012	China	84	clear cell renal cell carcinoma	qRT-PCR	>0.0362	TTP	7
Liu WF^28^	2012	China	144	gastric cancer	IHC	mild staining cells >10% of tumor cells	OS	6
Wang W^13^	2011	China	67	gallbladder adenocarcinoma	IHC	positive cells ≥25% and/or scores ≥2	OS	7
Zabouo G^29^	2009	France	635	breast cancer	IHC	scores of 2+ and 3+	OS	8
Aldovini D^30^	2006	Italy	126	epithelial ovarian cancer	IHC	score greater/equal to 2 (range of 0–7)	OS, TTP	8
Kristiansen G^25^	2003	Germany	38	lung adenocarcinoma	IHC	score of 2+	OS	6

ICH: Immunohistochemistry; qRT-PCR: quantitative Real-time polymerase chain reaction.

### Statistical analysis

The data collected from each eligible article was used to evaluate the relations between CD146 expression and solid cancer prognosis by meta-analysis. Pooled HRs and 95% CIs for these outcome endpoints (OS, TTP) were calculated. Subgroup analysis was performed when there were at least three studies in each subgroup. Statistical heterogeneity was assessed using the Q test, and a P value > 0.10 suggested a lack of heterogeneity among studies. We also quantified the effect of heterogeneity using *I* ^2^ = 100% × (Q − df)/Q. *I* ^2^ values of <25% may be considered “low”, values of about 50% may be considered “moderate” and values of >75% may be considered “high”^33^. According to the absence or presence of heterogeneity, random effects model or fixed effects model was used to merge the HR, respectively. Without statistical heterogeneity, a fixed effects model was employed to calculate the pooled HRs, otherwise random effects model was used^34^. Funnel plots and the Egger’s test were used to estimate the possible publication bias^35^. If a publication bias did exist, the Duval and Tweedie’s trim and fill method was then used to assess its influence on the overall effect^36^. Sensitivity analysis was also conducted to find out if certain individual article could influence the overall result. The Stata 14.0 (StataCorp, College Station, TX) was used to conduct statistical analyses. P values for all comparisons were two-tailed and statistical significance was defined as p < 0.05 for all tests, except those for heterogeneity.

## Results

### Demographic characteristics

**Figure 1:**
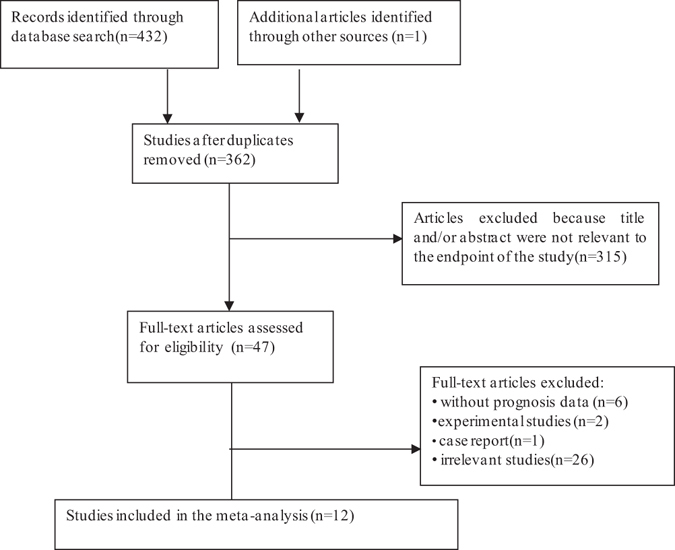
The flow chart of the selection process in our meta-analysis.

Through a literature search of the PubMed, Embase, and Web of Science databases, a total of 432 articles were retrieved, using different combinations of key terms, one additional article identified by scanning the reference of the selected studies As showed in the search flow diagram (Fig. 1), 432 records were originally acquired using the predefined search strategy. Owing to repeated data, 71 records were removed. After glancing over the retrieved titles and abstracts, 315 records were excluded because of no relevant endpoint provided. The rest of 47 records were downloaded with full-text and carefully retrieved one by one. Among them, 35 studies were eliminated, including two studies that were experimental study, one was case report, and six without prognosis data, 26 were unrelated. Consequently, 12 published studies counting 2,694 patients that up to the inclusion norm were finally elected for the meta-analysis, which were then used to assess the relevance between CD146 expression and solid tumor prognosis. The median sample-size of these testers was 118, with a wide range from 36 to 1080. Among all cohorts, Mongoloid (n = 9) became the major race of literatures, followed by Caucasian (n = 3). As for the cancer type, one study evaluated hepatocellular carcinoma, one study evaluated uterine leiomyosarcoma, one study evaluated esophageal squamous cell carcinoma, there studies evaluated lung cancer, one study evaluated colorectal cancer, one study evaluated clear cell renal cell carcinoma, one study evaluated gastric cancer, one study evaluated gallbladder cancer, one study evaluated breast canser, one study evaluated epithelial ovarian cancer. Overall, 11 studies focused on OS, four studies focused on TTP.

### Evidence synthesis

**Figure 2:**
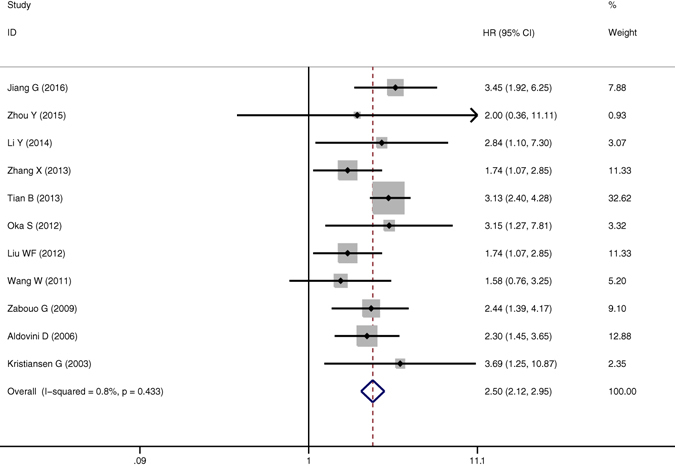
The correlation between CD146 expression and overall survival (OS) in solid tumors.

**Figure 3:**
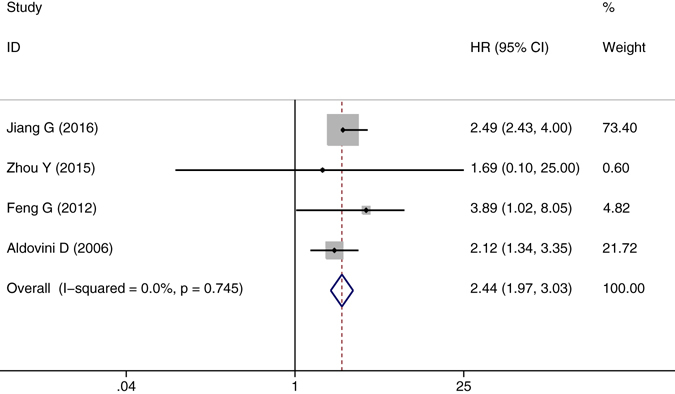
The correlation between CD146 expression and time to progression (TTP) in solid tumors.

The meta-analysis of CD146 expression was established in two outcome endpoints: OS, TTP. 11 articles were involved in OS of the meta-analysis. A fixed effects model was utilized to calculate the pooled hazard ratio (HR) and 95% confidence interval (CI) due to the heterogeneity test testified a P value of 0.433 and an *I* ^2^ values of 0.8%. The results suggested that CD146 overexpression was related with poor OS of solid tumors (pooled HR = 2.496, 95% CI = 2.115–2.946, P = 0.000) (Fig. 2). Four studies were included in TTP of the meta-analysis. On account of the heterogeneity test reported a P value of 0.745 and an *I* ^2^ values of 0.0%, a fixed-effects model was then used. The results showed a significant association between CD146 expression and TTP (pooled HR = 2.445, 95% CI = 1.975–3.027, P = 0.000) (Fig. 3). Subgroup study was then performed, the results suggested that the associations between CD146 overexpression and poor OS and poor TTP were significant in Mongoloid patients (OS: pooled HR = 2.508, 95% CI 2.073–3.034, *P* < 0.001; TTP: pooled HR = 2.544, 95% CI = 1.998–3.238, *P* = 0.000), as well as in Caucasian (OS: pooled HR = 2.461, 95% CI 1.759–3.444, *P* < 0.001). The significant correlation was also spotted between CD146 overexpression and poor OS in patients with lung cancer (OS: pooled HR = 2.172, 95% CI = 1.453–3.246, *P* < 0.001) and digestive system cancer (pooled HR = 2.661, 95% CI = 2.149–3.295, *P* < 0.001).

### Publication bias and sensitivity analysis

**Figure 4:**
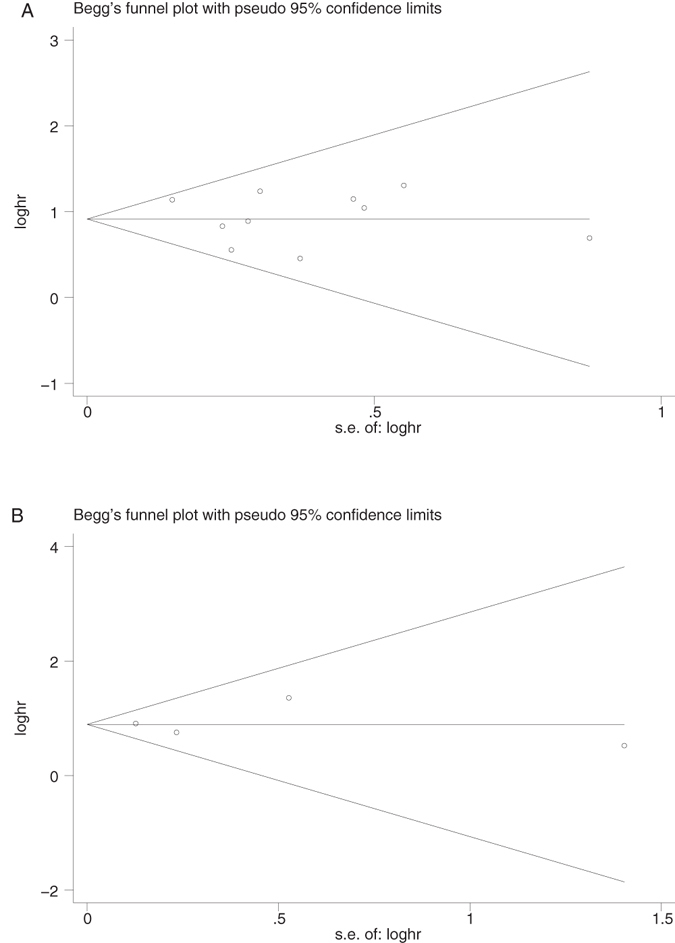
Begg’s funnel plots for the studies involved in the meta-analysis. (**A**) Overall survival. (**B**) Time to progression (TTP). Abbreviations: loghr, logarithm of hazard ratios; s.e., standard error.

**Figure 5:**
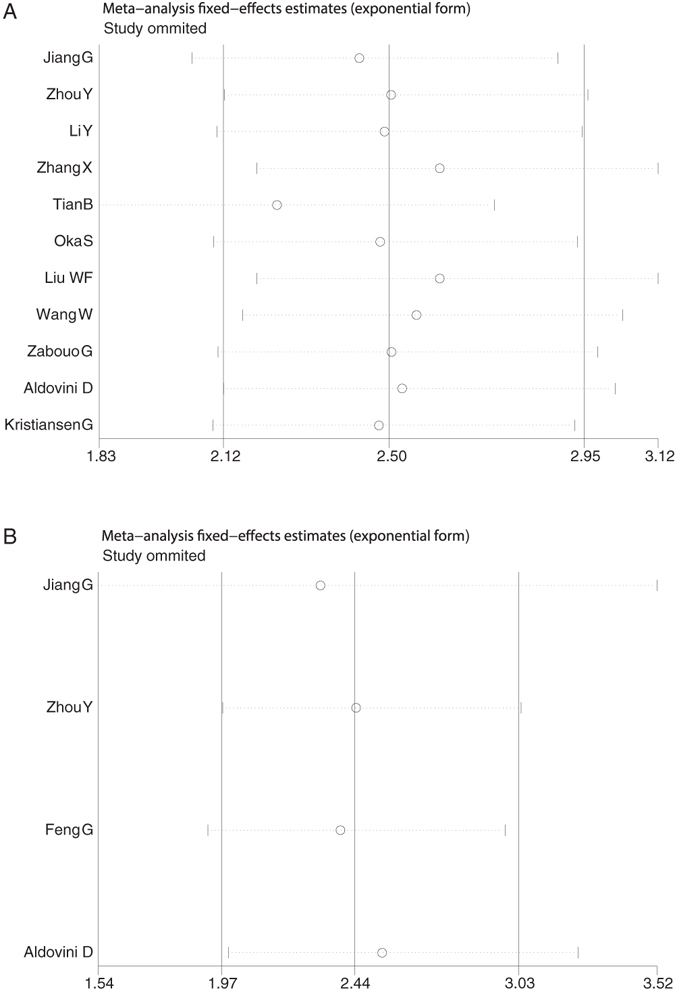
Sensitivity analysis of the meta-analysis. (**A**) Overall survival. (**B**) Time to progression (TTP).

Begg’s funnel plot and Egger’s test were utilized to evaluate the publication bias of these involved literatures. As shown in (Fig. 4), the shapes of the funnel plots for the OS and TTP showed no evidence of obvious heterogeneity, and Egger’s tests revealed no publication bias regarding both OS (P = 0.636) and TTP (P = 0.887). Sensitivity analyses were further done to determine the sturdiness of the results depicted above. No single study controlled this meta-analysis, and removal of any individual study had no significant effect on the overall conclusion (Fig. 5).

## Discussions

Overexpression of CD146 had been reported to promote cancer progression and predict poor prognosis of cancer patient. Many clinical studies investigated the prognostic value of CD146 over-expression. Nevertheless, most of these studies, including limited number of patients, remain have incomprehensive conclusions. This current meta-analysis is the first complete overview of all published clinical studies exploring the impact of CD146 expression on prognosis of many solid tumors.

We scientifically estimated survival data of 2,694 solid tumor patients included in 12 different studies. In general, these results clearly indicated that high CD146 expression was a poor prognostic factor in solid tumors, with both results of poor OS (pooled HR = 2.496, 95% CI = 2.115–2.946, P = 0.000) and poor TTP (pooled HR = 2.445, 95% CI = 1.975–3.027, P = 0.000). Similarly, subgroup analysis revealed the associations between CD146 overexpression and poor OS were significant within Mongoloid and Caucasian, and poor TTP within Mongoloid. When data was stratified according to cancer types, the results showed the prognostic value of CD146 over-expression was significant in digestive system neoplasms and lung cancer.

As far as we known, this present study is the first and most all-sided meta-analysis systematically discovering the possible prognostic role of CD146 up-regulation in solid tumors. Our assessable results strongly reinforced the current mainstream perspective that an adverse impact of CD146 redundancy was associated with the OS and TTP. Also, several important insinuations in this meta-analysis were presented. First, high CD146 expression may be a common poor prognostic marker in solid tumors. In this meta-analysis, we incorporated ten different cancer types, including hepatocellular carcinoma^20^, leiomyosarcoma^21^, esophageal squamous cell carcinoma^22^, lung cancer^23–25^, colorectal cancer^26^, clear cell renal cell carcinoma^27^, gastric cancer^28^, gallbladder adenocarcinoma^13^, breast cancer^29^, epithelial ovarian cancer^30^. The pooled results from these cancer types confirmed that high CD146 expression was associated with poor OS and TTP, and this finding can be extended to all solid tumors. Second, we verified that high CD146 expression associated with poor OS in Mongoloid and Caucasian patients, as well in digestive system neoplasms and lung cancer, and TTP in Mongoloid patients. Finally, it underlines the potential to develop CD146 as a valuable therapeutic target and prognostic biomarker for solid tumors, though it needs to be further studied.

Aside from the inspiring outcomes, limitations still lay in this assessable meta-analysis. First of all, most of the included studies were designed as retrospective studies, and such studies were more likely to be published if they have positive results than if which have negative one. Furthermore, the method assessing CD146 expression and defining CD146 positivity were inconsistent. Consequently, our estimation of the associations between overexpression of CD146 and outcomes may have been overestimated.

To sum up, high CD146 expression in solid tumor tissues association with poor survival was clearly demonstrated in the present meta-analysis. We suggest that CD146 may be a useful prognostic biomarker, but if it would be a promising therapeutic target for solid tumors still need to be ulteriorly researched. Furthermore, further studies related to specific tumor types and perspectives are required to corroborate the clinical utility of CD146 expression in solid tumors.
